# Rapamycin rescues mitochondrial dysfunction in cells carrying the m.8344A > G mutation in the mitochondrial tRNA^Lys^

**DOI:** 10.1186/s10020-022-00519-z

**Published:** 2022-08-03

**Authors:** Mariantonietta Capristo, Valentina Del Dotto, Concetta Valentina Tropeano, Claudio Fiorini, Leonardo Caporali, Chiara La Morgia, Maria Lucia Valentino, Monica Montopoli, Valerio Carelli, Alessandra Maresca

**Affiliations:** 1 IRCCS Istituto delle Scienze Neurologiche di Bologna, Programma di Neurogenetica, via Altura 3, 40139 Bologna, Italy; 2 Department of Biomedical and NeuroMotor Sciences, University of Bologna, via Altura 3, 40139 Bologna, Italy; 3 Department of Pharmaceutical and Pharmacological Sciences, University of Padova, via Largo Meneghetti 2, 3513 Padova, Italy

**Keywords:** MERRF, Mitochondrial DNA, Niacin, PGC-1α, Rapamycin, mTORC1, Mitochondrial dysfunction, Mitochondrial biogenesis

## Abstract

**Background:**

Myoclonus, Epilepsy and Ragged-Red-Fibers (MERRF) is a mitochondrial encephalomyopathy due to heteroplasmic mutations in mitochondrial DNA (mtDNA) most frequently affecting the *tRNA*^*Lys*^ gene at position m.8344A > G. Defective tRNA^Lys^ severely impairs mitochondrial protein synthesis and respiratory chain when a high percentage of mutant heteroplasmy crosses the threshold for full-blown clinical phenotype. Therapy is currently limited to symptomatic management of myoclonic epilepsy, and supportive measures to counteract muscle weakness with co-factors/supplements.

**Methods:**

We tested two therapeutic strategies to rescue mitochondrial function in cybrids and fibroblasts carrying different loads of the m.8344A > G mutation. The first strategy was aimed at inducing mitochondrial biogenesis directly, over-expressing the master regulator PGC-1α, or indirectly, through the treatment with nicotinic acid, a NAD^+^ precursor. The second was aimed at stimulating the removal of damaged mitochondria through prolonged rapamycin treatment.

**Results:**

The first approach slightly increased mitochondrial protein expression and respiration in the wild type and intermediate-mutation load cells, but was ineffective in high-mutation load cell lines. This suggests that induction of mitochondrial biogenesis may not be sufficient to rescue mitochondrial dysfunction in MERRF cells with high-mutation load. The second approach, when administered chronically (4 weeks), induced a slight increase of mitochondrial respiration in fibroblasts with high-mutation load, and a significant improvement in fibroblasts with intermediate-mutation load, rescuing completely the bioenergetics defect. This effect was mediated by increased mitochondrial biogenesis, possibly related to the rapamycin-induced inhibition of the Mechanistic Target of Rapamycin Complex 1 (mTORC1) and the consequent activation of the Transcription Factor EB (TFEB).

**Conclusions:**

Overall, our results point to rapamycin-based therapy as a promising therapeutic option for MERRF.

**Supplementary Information:**

The online version contains supplementary material available at 10.1186/s10020-022-00519-z.

## Background

Fukuara and colleagues described in 1980 a new syndrome characterized by “dyssynergia cerebellaris myoclonica” and the hallmark of mitochondrial myopathy with Ragged-Red-Fibers (RRFs) (Fukuhara et al. 1980). This syndrome is now known by the acronym MERRF, which stands for Myoclonus, Epilepsy, and RRF, and is one of the major syndromic phenotypes grouped under the denomination of mitochondrial encephalomyopathies (Carelli and La Morgia 2018). The pathological and biochemical features were better defined (Wallace et al. 1988; Berkovic et al. 1989) just before the discovery of the mitochondrial DNA (mtDNA) heteroplasmic mutation m.8344A > G/*MT-TK* in the *tRNA*^*Lys*^ gene, which still remains the most frequent genetic cause of MERRF (Shoffner et al. 1990).

As for other mtDNA mutations, the initial clinical spectrum expanded to include some peculiar phenotypes such as symmetrical lipomatosis, spinocerebellar-like ataxia and the severe Leigh syndrome, affecting a subset of patients carrying the MERRF mutation (Holme et al. 1993; Howell et al. 1996; Monden et al. 2013). More recent investigations of large cohorts of patients carrying the m.8344A > G/*MT-TK* mutation brought to redefine the core symptoms of most patients as myoclonic ataxia, with prevalent cerebellar involvement, whereas only a limited subset had the full-blown MERRF phenotype (Mancuso et al. 2013). Other frequent symptoms may include sensorineural deafness, CPEO and myopathy, as well as optic atrophy (Mancuso et al. 2013; Altmann et al. 2016). Over the years, also the landscape of mtDNA mutations associated with MERRF widened, implicating different changes either affecting the tRNA^Lys^ (Silvestri et al. 1992; Ozawa et al. 1997), or other tRNAs such as tRNA^Ser^ (Tiranti et al. 1995; Jaksch et al. 1998), typically associated with deafness.

Many studies on the most frequent m.8344A > G/*MT-TK* mutation, by studying available patient-derived tissues, highlighted a critical threshold necessary to manifesting symptoms (Boulet et al. 1992) and the correlation of heteroplasmic mutational load with clinical phenotypes (Larsson et al. 1992; Hammans et al. 1993).

Furthermore, since its identification, the MERRF mutation has also been investigated by using the cybrid cell model, documenting a global impairment in translation of the 13 mtDNA-encoded proteins, characterized by the presence of some aberrant translation products (Chomyn et al. 1991, 1994). Further studies deepened the understanding of the pathogenic mechanism showing altered tRNA^Lys^ amino-acylation and premature termination of translation (Enriquez et al. 1995), as well as a wobble base modification defect (Yasukawa et al. 2001). A derangement of mitochondrial Ca^2+^ homeostasis has also been documented in MERRF, with normal cytosolic Ca^2+^responses (Brini et al. 1999).

Concerning therapeutic options, the symptomatic management of myoclonic epilepsy with frequent photoinduced seizures remains a priority and follows the guidelines for myoclonic epilepsies (Shahwan et al. 2005), as well as supportive measures targeting muscle weakness with a combination of co-factors/supplements (Rodriguez et al. 2007). At the preclinical level, treatment of MERRF cells with drugs affecting organellar Ca^2+^ transport mostly restored both the agonist-dependent mitochondrial Ca^2+^ uptake and the ensuing stimulation of ATP production (Brini et al. 1999). Some indications of Coenzyme Q effectiveness have been also provided by in vitro studies (Mata et al. 2012). Moreover, in vitro correction of defective tRNA aminoacylation by the Cterm of human mt-leucyl tRNA synthetase has shown to be effective also for the classical MERRF mutation (Perli et al. 2016, 2020), as well as molecular approaches aimed at heteroplasmy shifting (Hashimoto et al. 2015; Gammage et al. 2016). These are all promising strategies under development for MERRF, with various levels of proof of principle in cell and animal models, but despite these efforts clinical management of MERRF patients remains highly problematic.

To speed therapeutic options for MERRF, we tested in vitro different pharmacological approaches aimed at manipulating the control of mitochondrial biogenesis and removal (Cerutti et al. 2014; Khan et al. 2014; Civiletto et al. 2018), with the final goal of shifting heteroplasmy or setting a compensatory strategy. To this end we used drugs suitable to be repurposed for rapid translation into approved compassionate use in single patient clinical trial.

## Methods

### Cell lines and culture conditions

Fibroblast cell lines were established from skin biopsies, after having obtained informed and written consent from patients and controls for the study and for all procedures. MERRF fibroblasts were generated from three patients (P1, P2 and P3) carrying the m.8344A > G mutation at different heteroplasmy (Additional file 1: Fig S1B) and compared with age and sex-matched fibroblasts derived from three healthy donors. Control fibroblasts carried mtDNA haplogroups T, U5a and U5b, whereas MERRF fibroblasts carried haplogroups H (P1 and P2) and U5b (P3). Fibroblasts derived from P1, carrying intermediate loads of m.8344A > G mutation, underwent a spontaneous and gradual loss of mutant mtDNA during cell culturing, resulting in a shift of heteroplasmy below the threshold for the pathogenic phenotype. Consequently, it was not possible to have complete set of experiments (biological triplicates) for this cell line for the pharmacological treatments, as well as testing the PGC1-α overexpression, as detailed in the figure legends.

Transmitochondrial cytoplasmic hybrids (cybrids) were generated using skin fibroblasts derived from P2 and the 143B.TK^−^ osteosarcoma cell line, as previously described (King and Attardi 1996). Three different clones were used for the experiments, one with intermediate and two with high loads of MERRF mutation, together with two different haplogroup-matched WT cybrid lines.

Control and mutants cell lines are cultured in Dulbecco Modified Eagle Medium (DMEM, GIBCO) supplemented with 10% fetal bovine serum (FBS, GIBCO), 2 mmol/l L-glutamine, 100 units/ml penicillin and 100 μg/ml streptomycin, at 37 °C in a 5% CO_2_ humidified incubator. Culture medium of mutant cybrid cell lines are also supplemented with 50 μg/ml uridine. To assess cellular response to nicotinic acid (NA), cells were treated with 10 mM NA (Sigma Aldrich, N0761) (or DMSO, as vehicle) for 96 h, an experimental condition previously tested in human cells (Crowley et al. 2000). For mitochondrial respiration evaluation, after 72 h cells are seeded in the Seahorse 24-well plate and incubated with NA for other 24 h.

Rapamycin (Sigma-Aldrich, R0395) was administered for 96 h or chronically for 4 weeks at concentration of 20 nM, as previously reported (Dai et al. 2014).

### PGC-1α overexpression

The isoform 1 of human PGC-1α (NM_001330751) was cloned by PCR into the pLenti-DDK-P2A-Puro empty vector (OriGene Technologies, PS100092). Primer sequences are reported in Additional file 1: Primer List. Lentivirus particles containing either the empty plasmid or the PGC-1α plasmid were prepared using the Lentiviral Packaging Kit (OriGene, TR30037) following manufacturer’s instructions and used to infect the cell lines. In cybrids, stable PGC-1α overexpression has been performed selecting positive clones by puromycin resistance. Differently, transient PGC-1α overexpression has been carried out in fibroblasts, which have been analyzed after 72 h from the infection.

### Viability assay

Cell viability was assessed by colorimetric Sulforhodamine B (SRB) assay, after galactose-medium incubation. Briefly, cells were seeded in 24-well plate at concentration of 3 × 10^4^cell/well and, after 24 h, incubated in glucose-free DMEM supplemented with 5 mmol/L galactose, 5 mmol/L Na-pyruvate, and 10% FBS (DMEM–galactose). Adherent cells were then fixed at 0 h, 24 h, 48 h and 72 h with 10% (final concentration) trichloroacetic acid (MERCK, T6399) for 1 h at 4 °C and stained with 0.4% SRB (MERCK, S1402) in 1% acetic acid. After 30 min of incubation, wells were washed with 1% acetic acid and the dye was solubilized with 10 mM Tris pH 10.5. Absorbance at 564 nm was then acquired by Enspire microplate reader instrument (Perkin Elmer).

### Western blot and antibodies

Total lysates were prepared using RIPA lysis buffer and a standard protocol. Proteins were separated on pre-cast NuPAGE 4–12% and 12% bis–tris glicyne gels (Life Technologies) and then transferred on nitrocellulose membranes, using the XcellSure Lock (Life Technologies) apparatus. After blocking with 5% milk, membranes were blotted with primary antibodies specific for OXPHOS cocktail (Abcam AB110411, 1:500), ACTIN (Abcam AB179467, 1:5000), TOM20 (CST 42,406, 1:1000), TIM23 (BD 611,222, 1:2000), VDAC (Abcam AB14734, 1:2000), TFAM (Abcam AB47517, 1:1000), PGC-1α (Calbiochem ST1202, 1:500), S6 (CST 2217, 1:1000), S6-P 204/244 (CST 5364, 1:1000), LC3B (NovusBio NB100-2220, 1:1000). Fluorescent secondary antibodies anti-rabbit or anti-mouse (Licor, catalog 926–32,210 and 926–68,071, 1:5000) were used for immunodetection using the Odissey Fc instrument (Licor).

### Mitochondrial respiration evaluation

Oxygen consumption rate (OCR) was measured in adherent fibroblasts and cybrids (respectively 1,5 × 10^4^ and 4 × 10^4^ cells/well density) with the XFe24 Extracellular Flux Analyzer (Seahorse, Agilent Technologies). For mitochondrial respiration test, after baseline measurements, OCR was measured after sequentially adding oligomycin, carbonylcyanide 4-(trifluoromethoxy) phenylhydrazone (FCCP), rotenone and antimycin A to reach final concentrations of 1 μM. OCR was normalized to protein content, as cell number surrogate, measured by SRB assay, following standard protocol. Exclusively for rapamycin treatment, OCR was normalized by cell number count, to avoid possible bias in normalization due to suppression of protein synthesis after mTORC1 inhibition. Basal, ATP-linked and maximal respirations were calculated as previously described (Maresca et al. 2020).

### Mitochondrial DNA content and heteroplasmy assessment

Total DNA was isolated from fibroblasts using the NucleoSpin tissue kit (Machery & Nagel), following the manufacturers’ instructions. Mitochondrial DNA content was assessed by real time-PCR through absolute quantification, as previously described (Maresca et al. 2020).

Heteroplasmy assessment of the m.8344A > G mutation was performed by the SNaPshot assay (Cassandrini et al. 2006). Primer sequences are listed in Additional file 1: Primer List.

### Gene expression analysis

Total RNA was extracted from fibroblasts and cybrids using the Pure Link RNA mini kit (Ambion) and then treated with DNAseI enzyme (Sigma Aldrich). 1 μg of RNA was reverse transcribed by SuperScript VILO cDNA synthesis kit (Life Technologies) and analyzed by real time-PCR (LightCycler®480, Roche Diagnostics), using SYBR Green I- or Universal Probe Library (Roche Diagnostics)-based assays. Primer sequences are listed in Additional file 1: Primer List.

### Determination of NAD^+^ and NADH concentration

NAD^+^ and NADH concentrations were determined using a commercial kit based on colorimetric quantification (MAK037; Sigma Aldrich). Briefly, 4 × 10^5^ cells for fibroblasts and 5 × 10^5^cells for cybrids were collected after 96 h of incubation with 10 mM NA or vehicle (DMSO) by centrifugation at 2500 × g and room temperature for 10 min. Pellets were washed once with PBS and resuspended in extraction buffer (260 uL for fibroblasts and 400 uL for cybrids), followed by 2 cycles of freeze/thawing on dry ice. Samples were clarified by centrifugation at 13,000 × g for 10 min and the supernatant was deproteinized by filtration through a 10-kDa cutoff spin filter (Millipore SAS). The assay was then performed according to the manufacturer’s instructions. Data were quantified using the optical density on EnSpire® Multimode Plate Reader (PerkinElmer). NADH concentrations were subtracted from the total NAD^+^ and NADH values to obtain NAD^+^, and levels were normalized to mg of proteins in the sample.

### Statistical analysis

Statistical analyses were performed using GraphPad Prism for Windows. Anova test (Tukey’s multiple comparisons test) was used to compare control means to individual MERRF mutant means. Unpaired two tail T-test was used to compare each “untreated” group to respective “treated” one. For all analyses, differences were considered significant at a *p value* ≤ 0.05.

## Results

### MERRF cell models display defective OXPHOS and altered mitochondrial protein expression

To recapitulate the mitochondrial dysfunction in cell models derived from MERRF patients, we characterized transmitochondrial cytoplasmic hybrids (cybrids) and fibroblasts carrying intermediate (50–60%) and high (80–90%) mutation loads (Additional file 1: Fig. S1A, S1B).

**Fig. 1:**
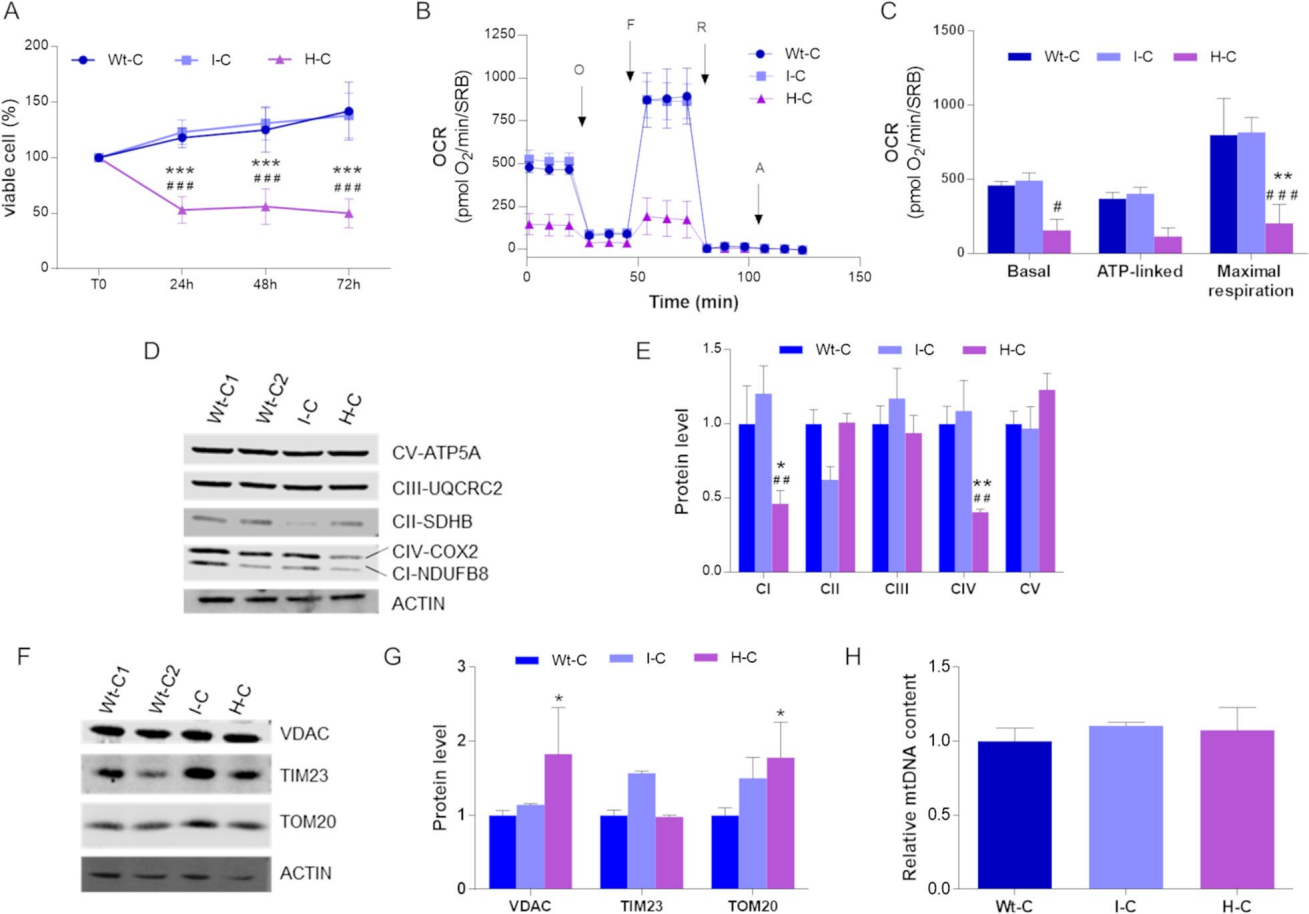
Bioenergetics assessment in MERRF cybrids.** A** Cell viability was evaluated growing cells for 24 h, 48 h and 72 h in galactose-medium and by performing SRB assay. Data are means ± SEM of four independent experiments (biological replicates). **B** OCR expressed as pmoles O_2_/min, normalized for protein content, under basal conditions and after injection of oligomycin (O), carbonyl cianide 4-(trifluoromethoxy) phenylhydrazone (FCCP; F), rotenone (R) and antimycin A (AA). Data are expressed as means ± SEM of three independent experiments (biological replicates). **C** Basal, ATP-linked and maximal respiration. All values are means and SEM of three independent experiments (biological replicates). **D** Western blot of OXPHOS subunits; ACTIN was used as loading control. A representative blot of three independent experiments, analyzing three biological replicates, is shown for each protein. **E** Densitometry of OXPHOS subunits of three independent experiments (biological replicates). All data are means and SEM and are normalized to control cells. **F** Western blot of VDAC, TIM23 and TOM20; ACTIN was used as loading control. A representative blot of three independent experiments (biological replicates) is shown for each protein. **G** Densitometry of VDAC, TIM23 and TOM20 content. All data are means and SEM and are normalized to control cells. **H** mtDNA content evaluation by qPCR. All values are expressed as means and SEM of three biological replicates and are normalized to control cells. Statistical analyses were performed with ANOVA test (Tukey’s multiple comparisons test). *, ** and *** values significantly different from the control cells, p < 0.05, p < 0.01, p < 0.001 respectively; ^#^, ^##^ and ^###^ values significantly different from the I-mutant, p < 0.05, p < 0.01, p < 0.001, respectively. *Wt-C* wild type cybrids, *I-C* intermediate heteroplasmy cybrids, *H-C* high heteroplasmy cybrids

When grown in galactose-containing medium, thus forcing cells to rely on oxidative phosphorylation (OXPHOS) to produce ATP, cybrids with high mutation load (herein named H-cybrids) exhibited a significant reduction in cell viability, whereas the intermediate mutation load cells (herein named I-cybrids) were not different from wild type (WT) cells (Fig. 1A). As an indicator of mitochondrial respiration, we assessed oxygen consumption rate (OCR) in live cells. According to the previous results on cell viability, OCR was dramatically reduced in H-cybrids, but not in I-cybrids, which showed a mitochondrial respiration comparable to that of WT cells (Fig. 1B, C). We investigated by Western blot the expression of various subunits of respiratory chain complexes evidencing a decrease of NDUFB8 (Complex I) and COX2 (Complex IV), exclusively in the H-cybrids (Fig. 1D, E). Similar results were observed after normalization for a mitochondrial matrix protein, citrate synthase (CS) (Additional file 1: Fig. S1C). As mitochondrial mass surrogate, we quantified structural proteins located in outer or inner mitochondrial membranes, showing a significant increase in the H-cybrids for VDAC and TOM20 (Fig. 1F, G). However, mtDNA content of MERRF cybrids was comparable to WT cells (Fig. 1H).

**Fig. 2:**
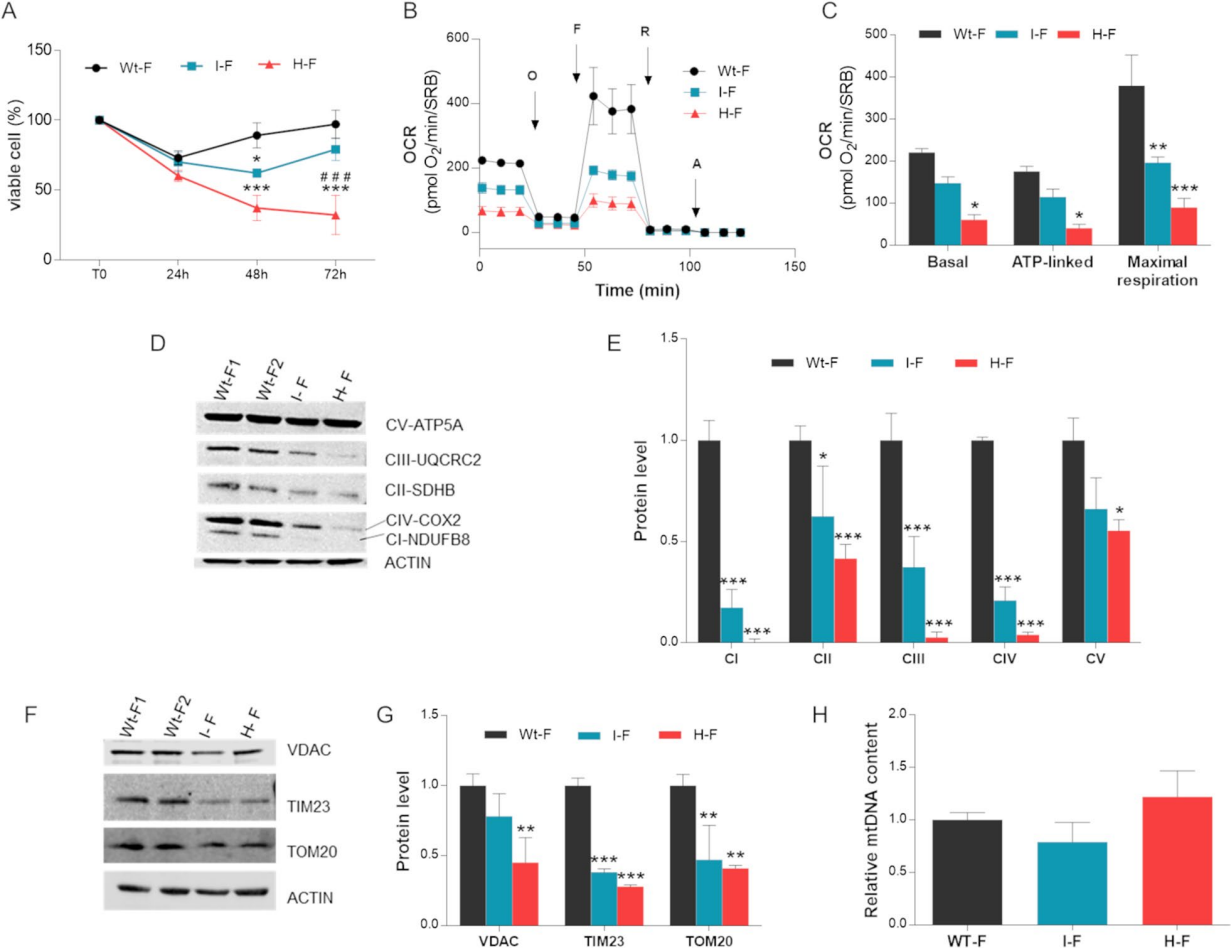
Bioenergetics assessment in MERRF fibroblasts.** A** Cell viability was evaluated growing cells for 24 h, 48 h and 72 h in galactose-medium and by performing SRB assay. Data are means ± SEM of three independent experiments (biological replicates). **B** OCR expressed as pmoles O_2_/min, normalized for protein content, under basal conditions and after injection of oligomycin (O), carbonyl cianide 4-(trifluoromethoxy) phenylhydrazone (FCCP; F), rotenone (R) and antimycin A (AA). Data are means ± SEM of three independent experiments (biological replicates). **C** Basal, ATP-linked and maximal respiration. All values are means + SEM of three independent experiments (biological replicates). **D** Western blot of OXPHOS subunits; ACTIN was used as loading control. One representative experiment out of three is shown. **E** Densitometry of OXPHOS subunits of three independent experiments (biological replicates). All data are means and SEM and are normalized to control cells. **F** Western blot of VDAC, TIM23 and TOM20; ACTIN was used as loading control. A representative blot of three independent experiments is shown for each protein. **G** Densitometry of VDAC, TIM23 and TOM20 content. All data are means and SEM and are normalized to control cells. **H** mtDNA content evaluation by qPCR. All values are expressed as means and SEM of four biological replicates and are normalized to control cells. Statistical analyses were performed with ANOVA test (Tukey’s multiple comparisons test). *, ** and *** values significantly different from the control cells, p < 0.05, p < 0.01, p < 0.001 respectively; ^#^, ^##^ and ^###^ values significantly different from the I-mutant, p < 0.05, p < 0.01, p < 0.001, respectively. *Wt-F* wild type fibroblasts, *I-F* intermediate heteroplasmy fibroblasts, *H-F* high heteroplasmy fibroblasts

To compare the two MERRF cell models, we performed the same analyses in patient-derived fibroblasts carrying intermediate (herein named I-fibroblasts) and high (herein named H-fibroblasts) mutation loads. At difference with the cybrid model, both MERRF fibroblasts were significantly less viable in galactose-medium than WT-fibroblasts, being H-fibroblasts the most compromised (Fig. 2A). Following the same trend, the H-fibroblasts had about 80% of reduction in basal, ATP-linked and maximal respiration, whereas the I-fibroblasts had a less severe defect with about 50% decrease in respiration (Fig. 2B, C). Again, both I-fibroblasts and H-fibroblasts presented a reduction of NDUFB8, SDHB (Complex II), UQCRC2 (Complex III), COX2 and ATP5A (Complex V) (Fig. 2D, E). After CS normalization, similar reduction was observed with the exception of a significant increase of CII and CV subunits in the I-fibroblasts (Additional file 1: Fig. S1D), possibly reflecting an attempt of these cells to compensate for the reduction of the other complexes. Moreover, MERRF fibroblasts had lower VDAC, TIM23, TOM20 protein levels (Fig. 2F, G), although mtDNA content was comparable to WT cells (Fig. 2H).

### Induction of mitochondrial biogenesis is not effective in rescuing the OXPHOS defect of MERRF cell models

Since the MERRF mutation is present exclusively in heteroplasmic state in tissues from patients, boosting mitochondrial biogenesis and increasing the absolute number of WT mtDNA molecules may have a beneficial effect on OXPHOS function. To verify this hypothesis, we induced mitochondrial biogenesis by either a pharmacological or a genetic approach.

As first strategy, we used nicotinic acid (NA) (also known as niacin or vitamin B3), one of the major precursors of the Nicotinamide Adenine Dinucleotide (NAD^+^) cofactor that is essential for several biological processes. Among these, NAD^+^ is used as substrate by SIRT1, which deacylates and activates the master regulator of mitochondrial biogenesis, the Peroxisome Proliferator Activated Receptor Coactivator 1 α (PGC-1α) (Koh and Kim 2021).

**Fig. 3:**
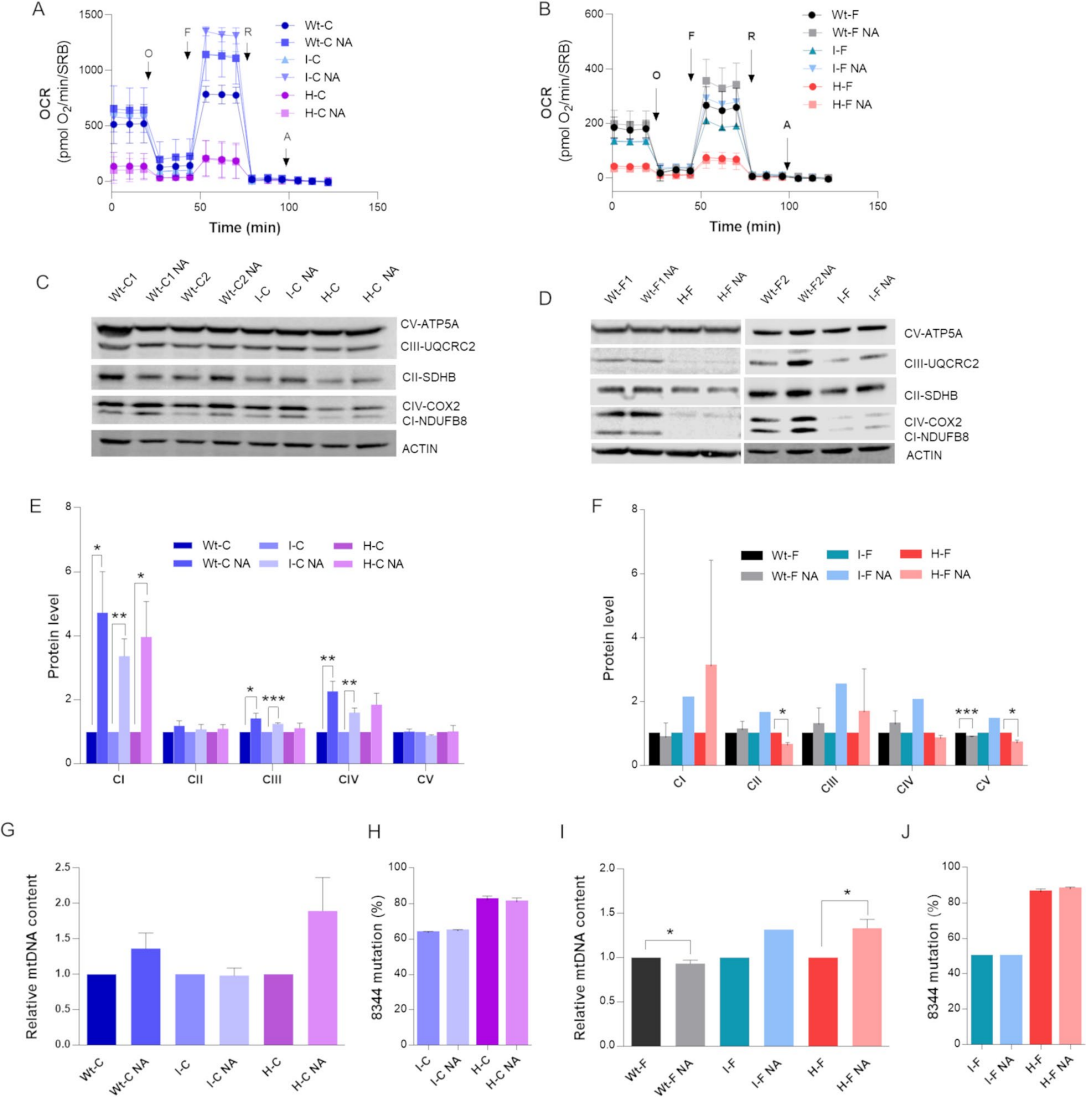
Nicotinic acid (NA) treatment in MERRF cybrids and fibroblasts. Cells are treated with vehicle (DMSO) or 10 mM NA for 96 h. **A**, **B** OCR in cybrids **A** and fibroblasts **B** after NA treatment; OCR expressed as pmoles O_2_/min and normalized for protein content, under basal conditions and after injection of oligomycin (O), carbonyl cianide 4-(trifluoromethoxy) phenylhydrazone (FCCP; F), rotenone (R) and antimycin A (AA). In cybrids, data are means ± SD of two experiments, analyzing two biological replicates. In fibroblasts, data are means ± SD of three experiments for Wt-F and of two experiments for H-F; only one experiment could be performed in I-F due to spontaneous heteroplasmy shift in culturing cells. **C**, **D** Western blot of OXPHOS subunits in cybrids **C** and fibroblasts **D**; ACTIN was used as loading control. A representative blot is shown for each protein. **E** Densitometry of OXPHOS subunits content in cybrids. Data of four independent experiments (biological replicates) are expressed as means and SEM, and are normalized to untreated cells. **F** Densitometry of OXPHOS subunits content in fibroblasts. Data are means and SD of three biological replicate for Wt-F and of two biological replicates for H-F; only one experiment could be performed in I-F due to spontaneous heteroplasmy shift in culturing cells; all data are normalized to untreated cells. **G** mtDNA content evaluation by qPCR in cybrids. All values are expressed as means and SEM of three biological replicates and are normalized to untreated cells. **H** Heteroplasmy level evaluation by snapshot method in cybrids. Values are expressed as means and SEM of three biological replicates. **I** mtDNA content evaluation by qPCR in fibroblasts. Data are means and SD of three biological replicates for Wt-F and of two biological replicates for H-F; only one experiment could be performed in I-F due to heteroplasmy shift in culturing cells. **J** Heteroplasmy level evaluation by snapshot method in fibroblasts. H-F values are expressed as means and SD of two biological replicates. Statistical analyses were performed using unpaired two-tail T-test. P value: * p < 0.05, ** p < 0.01, *** p < 0.001

MERRF cybrids and fibroblasts were incubated with NA 10 mM for 96 h and then analyzed for the respiratory capacity, expression of OXPHOS complexes, mtDNA content and heteroplasmy. An improvement in the maximal respiration was observed after NA treatment, although without reaching statistical significance and exclusively in WT cells and in cells carrying intermediate levels of the m.8344 mutation, both in cybrids and fibroblasts (Fig. 3A,B and Additional file 1: Fig. S1E, S1F). However, for the I-fibroblasts we were able to obtain only one valid experiment as this cell line underwent a shift of mtDNA heteroplasmy in favor of wild type molecules.

Similarly, NDUFB8 protein levels increased in WT- and mutant cybrids after NA treatment, as well as UQCRC2 and COX2 in WT- and I-cybrids (Fig. 3C, E). A similar trend was observed for I-fibroblasts, showing a rise in the expression of all OXPHOS proteins evaluated (Fig. 3D, F). Without reaching statistical significance WT- and H-fibroblasts also showed a variable increment in NDUFB8 (H-F), UQCRC2 (WT-F and H-F) and COX2 (WT-F) (Fig. 3D, F). Instead, ATP5A was significantly decreased in WT- and H-fibroblasts, as opposed to what observed for I-fibroblasts (Fig. 3D, F).

After NA treatment, a slight increase of mtDNA content occurred in WT- and H-cybrids and in H-fibroblasts, reaching statistical relevance in the latter only (Fig. 3G, I), yet maintaining the same heteroplasmic ratio (Fig. 3H, J). At difference, WT-fibroblasts exhibited reduced mtDNA content, which was statistically significant notwithstanding the limited magnitude (Fig. 3I). We also evaluated the effect of NA treatment on cellular NAD concentrations. In cybrids, NAD^+^ levels seems to be unchanged in treated cells compared to the NA treated, whereas NADH levels showed a slight increase, although not statistically significant (Additional file 1: Fig. S1G). In fibroblasts, both NAD^+^ and NADH were significantly increased, but exclusively in the H-fibroblasts (Additional file 1: Fig. S1H). It was not possible to measure NAD levels in the I-fibroblasts.

**Fig. 4:**
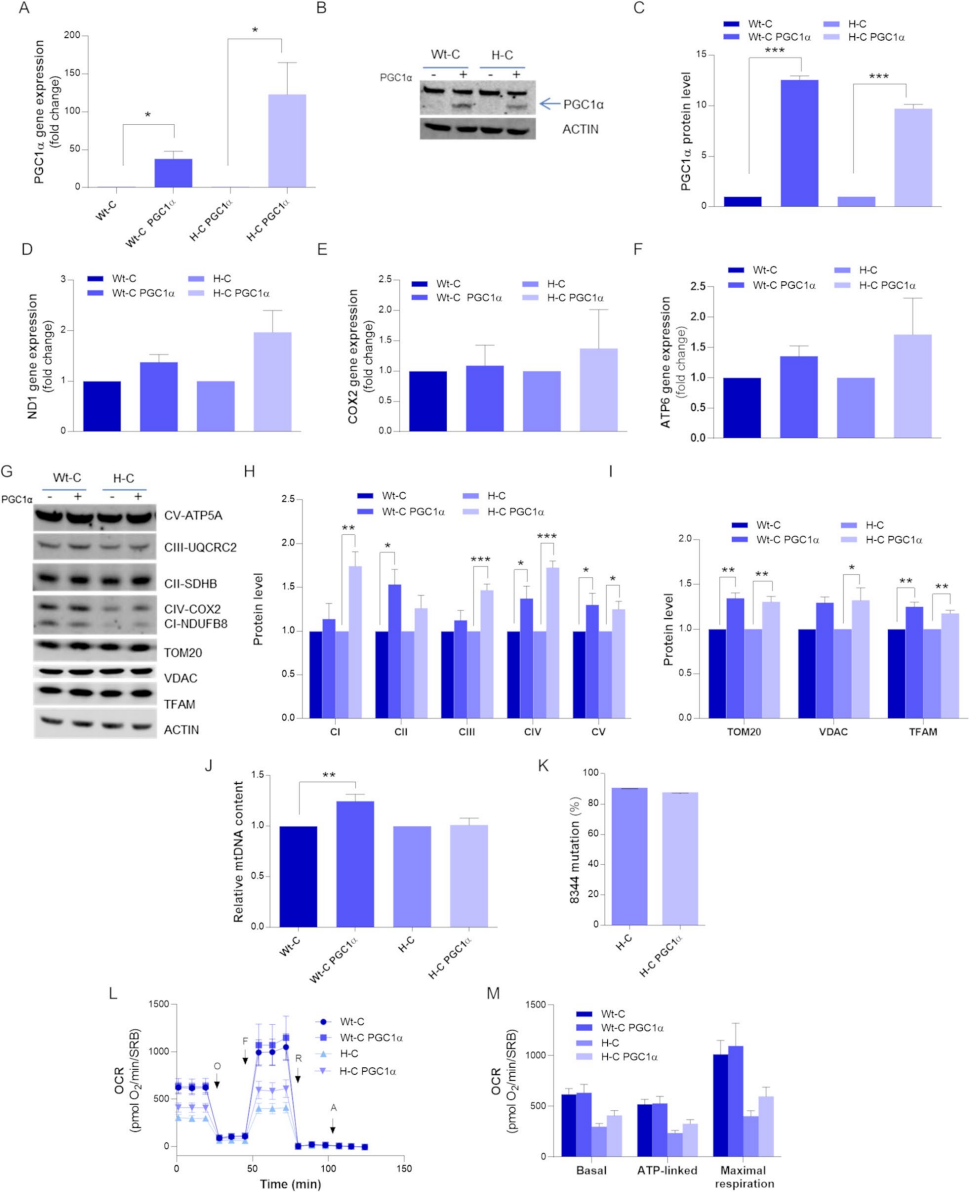
PGC-1α steady over-expression in MERRF cybrids. Wt-cybrids and H-cybrids were transduced with either an empty or a PGC-1α expressing lentiviral vector and then puromycin-selected to generate steady over-expressing PGC-1α cybrids lines. **A** PGC-1α gene expression was evaluated by qPCR. ACTIN was used as reference gene. **B** Western blot of PGC-1α; ACTIN was used as loading control. A representative blot of three independent experiments (biological replicates) is shown. **C** Densitometry of PGC-1α protein content, normalized to the cybrids transduced with the empty plasmid. **D** ND1, **E** COX2 and **F** ATP6 gene expression evaluated by qPCR. ACTIN was used as reference gene. Data are means and SEM of three biological replicate and are normalized to the cells transduced with the empty plasmid. **G** Western Blot of OXPHOS subunits and of TOM20, VDAC e TFAM protein. ACTIN was used ad loading control. One representative of three independent experiment (biological replicates) is shown. **H** Densitometric analysis of OXPHOS subunits protein level. Data, normalized to the cybrids transduced with the empty plasmid, are means and SEM of six independent experiments (biological replicates). **I** Densitometric analysis of TOM20, VDAC and TFAM mitochondrial mass proteins. Data are means and SEM of three independent experiments (biological replicates) and are normalized to the cells transduced with the empty plasmid. **J** mtDNA content evaluation by qPCR. All data are means + SEM of six biological replicates and are normalized to the empty cells. **K** m.8344A > G mutation heteroplasmy evaluation by snapshot method. Values are expressed as means and SEM of three biological replicates. **L** OCR expressed as pmoles O_2_/min normalized for protein content, under basal conditions and after injection of oligomycin (O), carbonyl cyanide 4-(trifluoromethoxy) phenylhydrazone (FCCP; F), rotenone (R) and antimycin A (AA). Data are means ± SEM of three independent experiments (biological replicates). **M** Basal, ATP-linked and maximal respiration were calculated from OCR traces and reported in the graph. All values are means and SEM of three independent experiments (biological replicates). Statistical analyses were performed using unpaired two-tail T-test. P value: *p < 0.05, **p < 0.01, ***p < 0.001

The second approach consisted in boosting mitochondrial biogenesis by overexpressing PGC-1α in both cybrid and fibroblast cell models. For cybrids this experiment was limited to the H-cybrids, as the I-cybrids did not show differences from WT, as reported above (Fig. 1). In H-cybrids, stable PGC-1α overexpression, verified by qPCR gene expression and western blotting (Fig. 4A-C), enhanced the expression of mitochondrial genes, as shown for ND1, COX2 and ATP6 genes (Fig. 4D–F), although without reaching a statistical significance. Congruently, cybrids overexpressing PGC-1α showed an increment of OXPHOS proteins, which was significant for NDUFB8 and UQCRC2 in H-cybrids, for SDHB in WT-cybrids, and for COX2 and ATP5A in both the cell lines (Fig. 4G, H). Structural proteins such as TOM20, VDAC and TFAM also increased after PGC-1α overexpression in the two cell lines (Fig. 4G, I), as well as mtDNA content displayed a similar rise in WT-cybrids (Fig. 4J). Furthermore, the heteroplasmic ratio in H-cybrids was unchanged (Fig. 4K). A slight improvement, not reaching statistical significance, in basal, ATP-linked and maximal respiration was observed in the H-cybrids after PGC-1α overexpression (Fig. 4L, M).

**Fig. 5:**
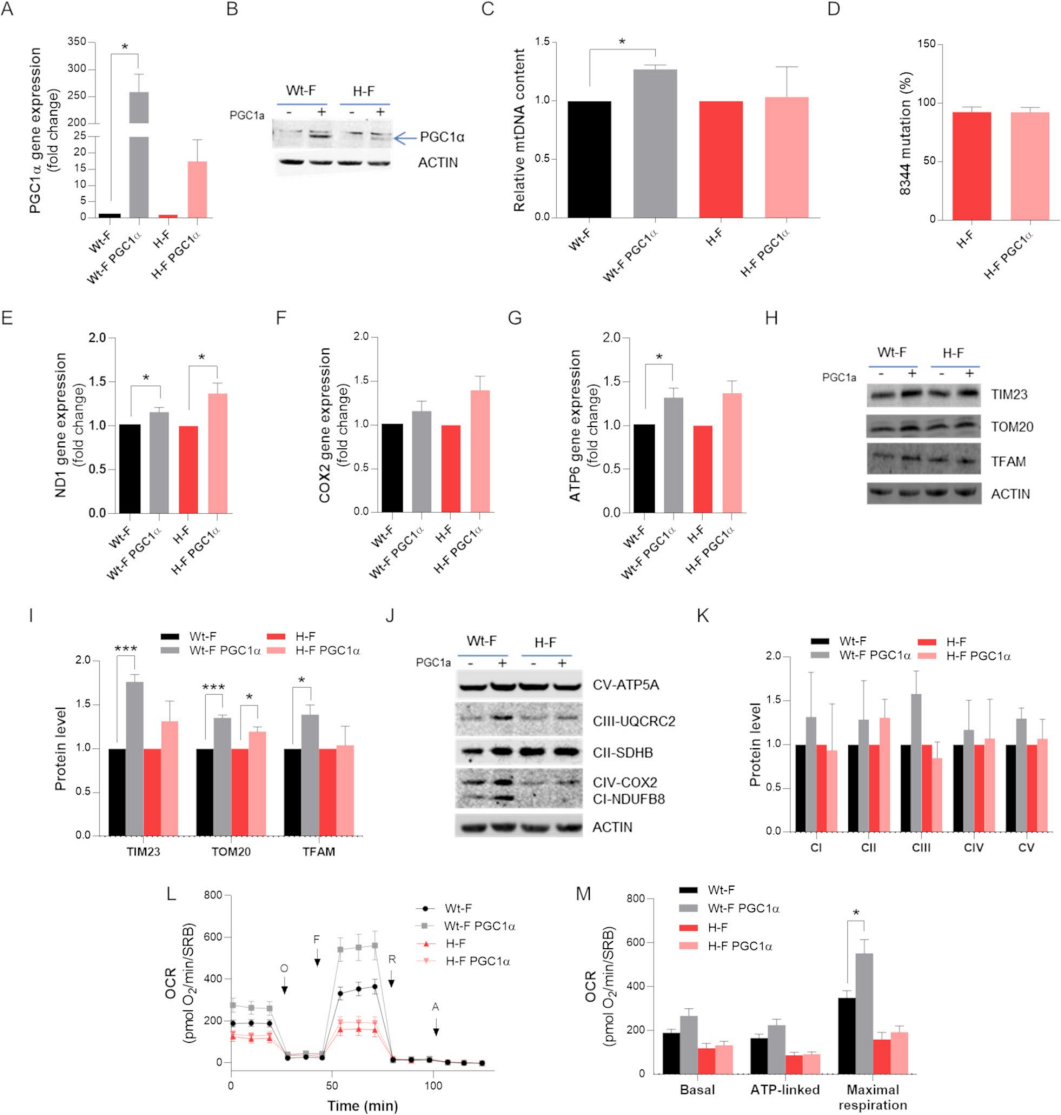
Transiently PGC-1α overexpression in MERRF fibroblasts. Wt-fibroblasts and H-fibroblasts were transiently transduced (72 h) with lentiviral vectors to generate either empty or over-expressing PGC-1α fibroblasts lines. **A** PGC-1α gene expression was evaluated by qPCR. ACTIN was used as reference gene. **B** Western blot of PGC-1α; ACTIN was used as loading control. A representative blot of three independent experiments (biological replicates) is shown. **C** mtDNA content evaluation by qPCR. **D** m.8344A > G mutation heteroplasmy evaluation by SNaPshot method. **E** ND1, **F** COX2 and **G** ATP6 gene expressions were evaluated by qPCR. ACTIN was used as reference gene. Data are means and SEM of three biological replicates and are normalized to the cells transduced with the empty plasmid. **H** Western Blot of TOM20, VDAC e TFAM mitochondrial mass proteins. ACTIN was used ad loading control. One representative of three independent experiment (biological replicates) is shown. **I** Densitometric analysis of TOM20, VDAC e TFAM mitochondrial mass protein. **J** Western Blot of OXPHOS proteins. ACTIN was used ad loading control. One representative of three independent experiment (biological replicates) is shown. **K** Densitometric analysis of OXPHOS subunits protein levels. **L** OCR expressed as pmoles O_2_/min normalized for protein content, under basal conditions and after injection of oligomycin (O), carbonyl cyanide 4-(trifluoromethoxy) phenylhydrazone (FCCP; F), rotenone (R) and antimycin A (AA). **M** Basal, ATP-linked and maximal respiration were calculated from OCR traces and reported in the graph. All data are means and SEM of three independent experiments, analyzing three biological replicates, and are normalized to cells transduced with the empty plasmid. Statistical analyses were performed using unpaired two-tail T-test. P value: * p < 0.05, ** p < 0.01, *** p < 0.001

In the fibroblast model we did not obtain stable transduction, therefore we performed only transient PGC-1α overexpression. Moreover, we had no longer available the I-fibroblasts for this analysis due to the heteroplasmy shift. Transient PGC-1α overexpression (Fig. 5A, B) significantly raised mtDNA content in the WT-fibroblasts, but no changes were observed in the H-fibroblasts for mtDNA levels and heteroplasmy (Fig. 5C, D). A slight increment in the ND1, COX2 and ATP6 gene expression was induced by PGC-1α overexpression (Fig. 5E–G), which translated into enhanced mitochondrial mass in the WT-fibroblasts and, at a lesser extent, in the H-fibroblasts (Fig. 5H, I). However, OXPHOS protein levels did not show any significant improvement (Fig. 5J, K), whereas mitochondrial respiration increased exclusively in the WT-fibroblasts (Fig. 5L, M).

These results suggest that induction of mitochondrial biogenesis per se may not be sufficient to recover the defective phenotype in MERRF models with high mutation loads.

### Mitochondrial respiration is rescued by rapamycin in fibroblasts carrying intermediate levels of MERRF mutation

We then tested a different therapeutic approach using rapamycin, which, according to most recent evidences, is able to orchestrate a more global metabolic rewiring acting on the mTOR complex 1 (mTORC1) regulation (Liu and Sabatini 2020). Moreover, under the assumption of increased removal of damaged mitochondria, possibly those with high mutation load, we also potentially envisaged a possible modulation of mutant mtDNA loads, ultimately shifting heteroplasmy in favor of wild-type mtDNA. In fact, rapamycin has been previously reported to drive selection against a pathogenic mtDNA mutation in cybrids carrying the heteroplasmic m.11778G > A/*MT-ND4* mutation, resulting in partial ATP levels restoration (Dai et al. 2014).

**Fig. 6:**
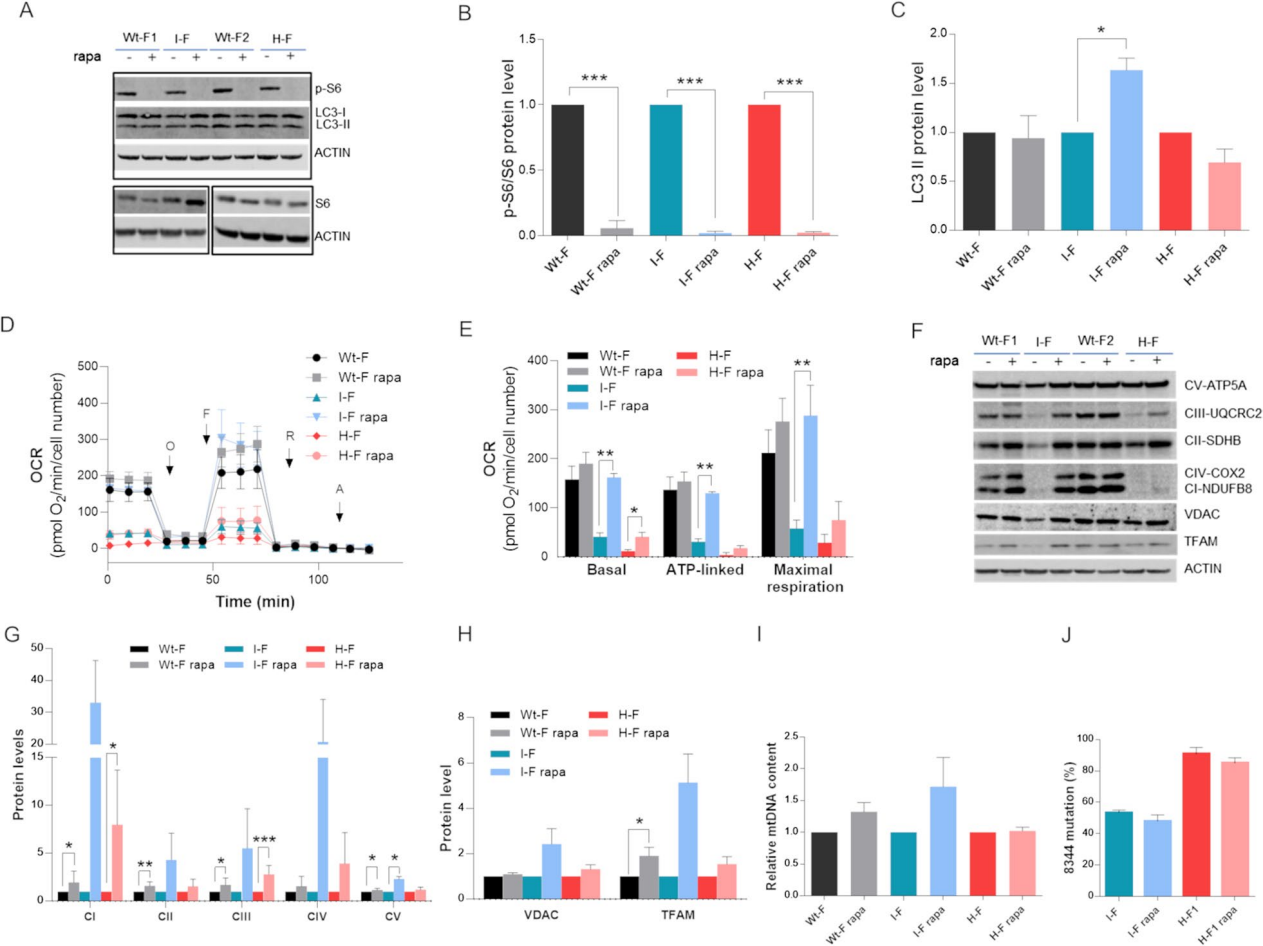
Mitochondrial respiration and mass assessment in MERRF fibroblasts treated with rapamycin. Wt-Fibroblasts, I-Fibroblasts and H-Fibroblasts were treated four weeks with 20 nM rapamycin. **A** Representative Western Blot of p-S6, S6 and LC3 proteins. ACTIN was used ad loading control. **B** Densitometric analyses of downstream target of mTORC1, p-S6 protein. **C** Densitometric analyses of LC3-II protein. **D** OCR expressed as pmoles O_2_/min normalized to cell number, under basal conditions and after injection of oligomycin (O), carbonyl cyanide 4-(trifluoromethoxy) phenylhydrazone (FCCP; F), rotenone (R) and antimycin A (AA). **E** Basal, ATP-linked and maximal respiration were calculated from OCR traces and reported in the graph. Data are means ± SEM of five experiments for Wt- and H-fibroblasts and of three experiments for I-fibroblasts. **F** Western Blot analyses of OXPHOS proteins, VDAC and TFAM mitochondrial mass proteins. One representative experiment is shown. Densitometric analyses of OXPHOS **G** and VDAC and TFAM proteins **H**. **I** mtDNA content evaluation by qPCR. **J** m.8344A > G mutation heteroplasmy evaluation by SNaPshot method. All data are normalized to untreated cells and, if not specifically indicated, are means and SD of six biological replicates for Wt-F, two biological replicates for I-F and four biological replicates for H-F. Statistical analyses were performed using unpaired two-tail T-test. P value: *p < 0.05, **p < 0.01, ***p < 0.001

Similarly, we administered rapamycin at a concentration of 20 nM for 4 weeks (Dai et al. 2014), comparing the effect of the treatment in WT-, I- and H-fibroblasts. Similar experiments on cybrids were not informative (Additional file 1: Fig. S2A). To verify the rapamycin-induced mTORC1 inhibition, we first assessed the phosphorylation of the S6 protein, a downstream target of mTORC1, demonstrating that phospho-S6 protein levels were consistently decreased after the rapamycin treatment (Fig. 6A, B). Although the proved inhibition of mTORC1 typically results in the stimulation of autophagy (Liu and Sabatini 2020), only the I-fibroblasts showed a relevant accumulation of the processed form of MAP1LC3B (herein named LC3-II), a well-known marker of autophagy, after the rapamycin treatment (Fig. 6A, C). However, the chronic treatment with rapamycin induced a significant increase of oxygen consumption in the I-fibroblasts, which completely rescued basal, ATP-linked and maximal respiration (Fig. 6D, E). A similar effect was observed also for the WT- and H-fibroblasts, with a significant increase for the latter of basal and ATP-linked respiration compared to the untreated cells (Fig. 6D, E). OXPHOS proteins and the markers for mitochondrial mass (VDAC and TFAM) were generally increased in both WT- and I-fibroblasts, whereas for H-fibroblasts this effect was negligible (Fig. 6F–H). MtDNA content showed the same tendency (Fig. 6I), and a slight decrease (about 5%) of mutant mtDNA molecules has been detected, however failing to reach a statistical significance (Fig. 6J).

**Fig. 7:**
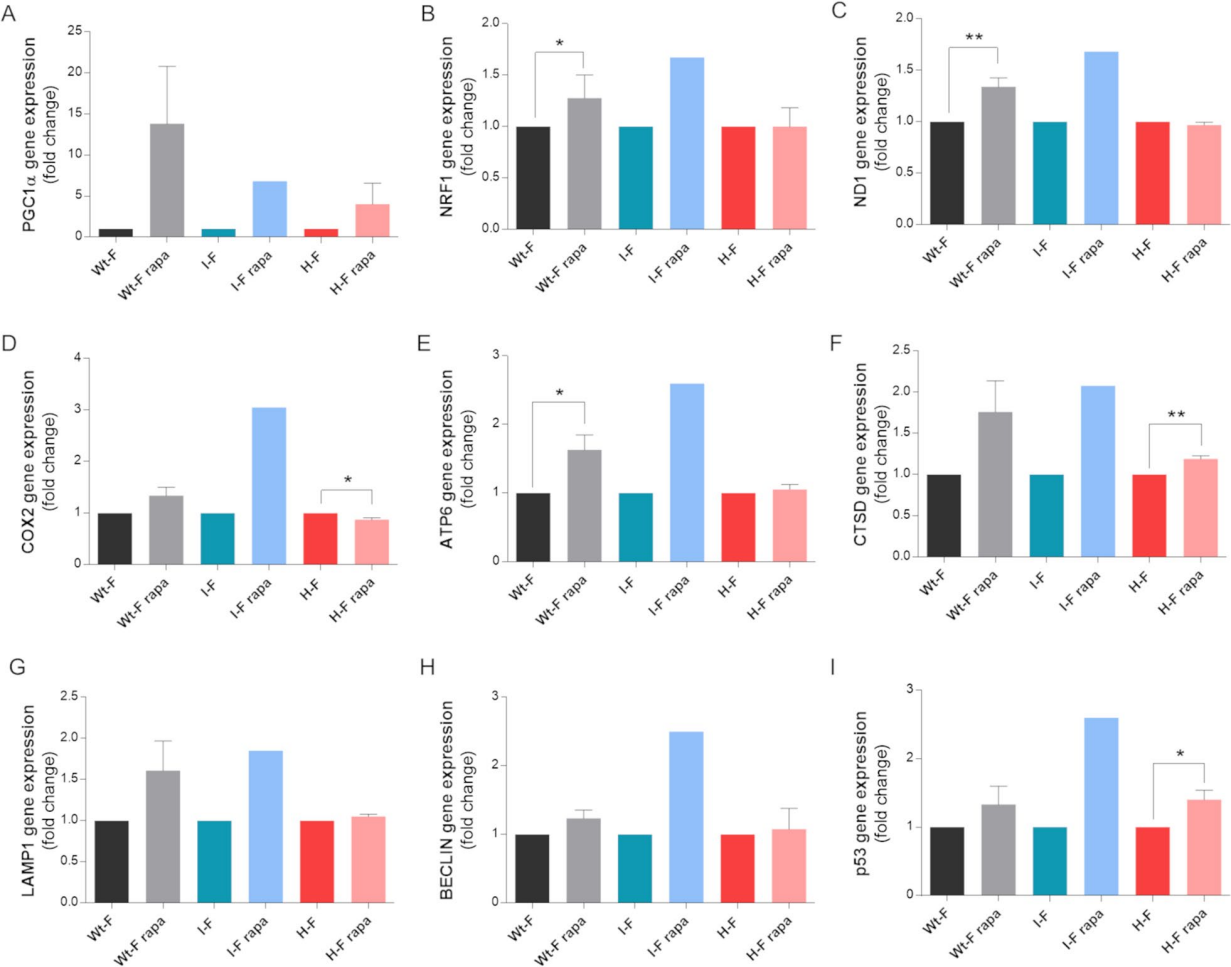
Gene expression of mitochondrial biogenesis and TFEB pathways in MERRF fibroblasts treated with rapamycin. Wt-fibroblasts, I-fibroblasts and H-fibroblasts were treated four weeks with 20 nM rapamycin. Gene expression of PGC-1α (**A**), NRF1 (**B**), ND1 (**C**), COX2 (**D**), ATP6 (**E**), CTSD (**F**), LAMP1 (**G**), BECLIN (**H**) and p53 (**I**) evaluated by qPCR. ACTIN was used as reference gene. Data are normalized to untreated cells and are means and SD of four biological replicates for Wt-F and three biological replicates for H-F. Only one experiment is reported for I-F. Statistical analyses were performed using unpaired two-tail T-test. P value: *p < 0.05, **p < 0.01, ***p < 0.001

To confirm the indications of activated mitochondrial biogenesis following this rapamycin treatment, we evaluated the gene expression of PGC-1α and NRF1, as transcriptional coactivators promoting mitochondrial gene transcription, and of a few mitochondrial genes. Consistent with the previous results, PGC-1α gene expression was induced by rapamycin in WT-, I- and H-fibroblasts (Fig. 7A), whereas NRF1 augmented exclusively in the WT and I-fibroblasts (Fig. 7B), coherently reflecting ND1, COX2 and ATP6 expression (Fig. 7C–E).

Among the downstream targets of mTORC1, the transcription factor EB (TFEB) is retained inactive in the cytoplasm through the phosphorylation executed by this complex. On the contrary, inhibition of mTORC1 promotes TFEB translocation into the nucleus initiating transcription of its target genes (Settembre et al. 2012; Martina et al. 2012). Besides its role in promoting lysosomal biogenesis and autophagy, TFEB has been recently implicated in mitochondrial quality control and in mitochondrial biogenesis (reviewed in (Wang et al. 2020). Thus, we tested for TFEB activation following the chronic rapamycin treatment evaluating the gene expression of four targets, namely Cathepsin D (CTSD), Lysosomal Associated Membrane Protein 1 (LAMP1), Beclin 1 (BECN1) and Tumor Protein 53 (P53). The mRNA levels resulted again elevated in the WT- and I-fibroblasts, without reaching statistical significance, whereas the H-fibroblasts showed an increment of CTSD and P53 only (Fig. 7F-I).

To verify if also an acute treatment with rapamycin could improve mitochondrial respiration, we administered rapamycin at the same concentration (20 nM) for 96 h to MERRF cells carrying high mutation loads (Additional file 1: Figs. S2 and S3). As observed with the 4 weeks treatment, H-cybrids did not respond to rapamycin (Additional file 1: Fig. S2B). Moreover, in this condition we did not observe any improvement in mitochondrial respiration or increase of OXPHOS proteins expression in H-fibroblasts, despite the significant inhibition of mTORC1 that, again, did not result in a significant increase of LC3-II protein levels (Additional file 1: Fig. S3A-G). This was accompanied by unchanged TFAM and VDAC protein levels, as well as mtDNA content, m.8344 mutation load, and PGC-1α and TFEB targets (CTSD, P53) gene expression (Additional file 1: Fig. S3F, Fig. S3H–M).

These results suggest that a chronic inhibition of mTORC1 may be effective in rescuing OXPHOS function in fibroblasts, particularly in those carrying intermediate levels of the MERRF mutation, through the combined action on lysosomal and mitochondrial biogenesis orchestrated by TFEB.

## Discussion

Our study, focused on testing rapidly transferable therapeutic options for MERRF, provides some important insights. Despite the general optimism on mitochondrial biogenesis as efficient compensatory strategy, both our pharmacological and genetic approaches, respectively with NA and PGC-1α overexpression, resulted substantially insufficient to produce a significant metabolic improvement in MERRF cells. This was particularly disappointing for high heteroplasmic mutant loads. Rapamycin instead led to promising reversal of bioenergetics cell impairment, which was essentially back to normal for the intermediate heteroplasmic loads in fibroblasts. Collaterally, we also documented substantial differences between the cybrid cell model and fibroblasts, as the MERRF mutation at intermediate heteroplasmic loads failed to reveal a phenotype in cybrids as opposed to a clear defect observed in fibroblasts. Furthermore, heteroplasmy was stable over time in cybrids, whereas we experienced sudden shifts to wild type mtDNA in fibroblasts. Overall, the current results underline the need of further mechanistic studies in cell models more reliably representing the target tissue of the disease, such as post-mitotic neuronal or muscle cells.

In the last three decades the mechanism leading from the MERRF point mutation to OXPHOS deficiency has been sufficiently well delineated. A severe defect in mitochondrial protein synthesis occurs due to the reduced steady-state levels of tRNA^Lys^ and its aminoacylation (Enriquez et al. 1995), as well as lack of post-transcriptional modifications, as the 5-taurinomethyl-2-thiouridine at the wobble position (Yasukawa et al. 2001) and the 1-methyladenosine at position m.8348 (Richter et al. 2018). Ultimately, a defective incorporation of lysine within the mtDNA-encoded proteins results in premature termination of translation and specific truncated protein products (Chomyn et al. 1991), but also instability of full-length proteins with increased rate of their degradation (Boulet et al. 1992; Richter et al. 2021). The final outcome is COX deficiency and reduced respiration rate, mitochondrial membrane potential and ATP synthesis (De la Mata et al. 2012; Chomyn 1998; James et al. 1996; Antonická et al. 1999). This mechanism is tightly related to the load of mutant mtDNA, with different thresholds identified depending on cell models or human-derived tissues (Boulet et al. 1992; Antonická et al. 1999; Zhou et al. 1997; Attardi et al. 1995).

Remarkably, we found a clear-cut difference between cybrids and fibroblasts with comparable heteroplasmy. Both H-cybrids and H-fibroblasts showed significantly reduced viability in galactose-containing medium and defective respiration capacity, concordant with previously published findings (De la Mata et al. 2012; Perli et al. 2016; James et al. 1996; Antonická et al. 1999; Attardi et al. 1995). Analysis of respiratory complexes and ATP synthase protein levels in H-cybrids revealed a significant reduction limited to CI and CIV subunits, at difference with H-fibroblasts that displayed a severe generalized reduction, although more marked for CI, CIII and CIV, which are notoriously assembled into the supramolecular structure defined as super-complex (Cogliati et al. 2021). This reduction was not restricted to the mitochondrial subunits, since, with the exception of CIV, all the other OXPHOS proteins evaluated are encoded by the nuclear genome. Notably, other nuclear-encoded mitochondrial proteins located in the OMM (VDAC, TOM20) or in the IMM (TIM23) were significantly reduced in H-fibroblasts, but normal or increased in H-cybrids, whereas mtDNA amount was comparable to controls in both models. As OXPHOS components, as well as TIM23, reside all on the IMM, our results may relate to architectural derangement of mitochondrial cristae, as previously well documented in MERRF (Brantová et al. 2006; Coquet et al. 1993; Kim et al. 2002), including the original disease description (Fukuhara et al. 1980). Alternatively, the production of aberrant proteins within mitochondria may induce retrograde signaling to the nucleus and activate a stress response, such as the *mitochondrial unfolded protein response* (mtUPR), ultimately leading to increased degradation of nuclear-encoded proteins targeted to mitochondria. This global proteostress has been previously demonstrated as consequence of defective mitochondrial tRNA taurine modification (Fakruddin et al. 2018) and may also occur in MERRF cells. Concordantly, two chemokines related to the mtUPR and the *mitochondrial integrated stress response* (mtISR), FGF21 and GDF-15 (Khan et al. 2017; Suomalainen and Battersby 2018), have been found increased in serum samples from MERRF patients (Maresca et al. 2020). The differences between cybrids and fibroblasts carrying comparable amount of mtDNA mutation most probably reside in the obviously different nuclear background and, consequently, in mitochondrial-nuclear crosstalk.

As far it concerns I-cybrids, these were unaffected in all parameters, whereas I-fibroblasts somehow were intermediate between H-fibroblasts and WT cells. Overall, the cybrid nuclear background compensates the intermediate heteroplasmy for MERRF mutation, possibly due to the cancerous nature of this osteosarcoma-derived cell line and its high reliability on glycolysis.

We then tested therapeutic strategies rapidly transferrable to patients, to fill the gap for this currently unmet burning need. Our first choice was to test the classic compensatory activation of mitochondrial biogenesis to efficiently complement the defective MERRF cells, as shown in other mitochondrial diseases (Viscomi and Zeviani 2020). Earlier work on cybrids carrying a mutation in the *tRNA*^*Leu(UUR)*^ gene associated to MELAS syndrome clearly highlighted, for example, that setting different mtDNA amounts could modulate the cell bioenergetics with the same mutant heteroplasmy, thanks to the absolute increase of the wild type mtDNA (Bentlage and Attardi 1996).

PGC-1α is one of the major regulators of the program leading to mitochondrial renewal, acting on transcription factors able to induce mtDNA transcription and translation. Its activity is finely tuned by post-translational modifications, mainly phosphorylation by AMPK and deacetylation by SIRT1 (Gureev et al. 2019). Among the different PGC-1α activators, precursors of the SIRT1 substrate NAD^+^, such as nicotinamide riboside or nicotinic acid, have been already tested in animal models of mitochondrial dysfunction (Cerutti et al. 2014; Khan et al. 2014). Moreover, a few clinical trials have been conducted, including one with nicotinic acid in patients with mitochondrial myopathy due to mtDNA single or multiple deletions that showed promising results (Viscomi and Zeviani 2020; Pirinen et al. 2020). In our hands, the treatment with nicotinic acid showed only negligible improvement of respiratory efficiency, limited to maximal respiration, and only in MERRF cells (both cybrids and fibroblasts) carrying intermediate heteroplasmy or in WT cybrids. Nicotinic acid boosted mitochondrial biogenesis in these cells, increasing OXPHOS subunits protein levels and mtDNA content. The most promising results were observed with I-fibroblasts, although derived from a single experiment due to the shift of mtDNA heteroplasmy. Disappointingly, although nicotinic acid increased mtDNA content in MERRF fibroblasts, in H-fibroblast this did not translate into significant increase of OXPHOS subunits, and, conversely, the treatment produced a decrease of CII and CV protein levels. Intriguingly, NAD^+^ levels resulted significantly increased only in the H-fibroblasts. A possible explanation may be that in cybrids and NAD^+^ is rapidly consumed or reduced in NADH, whereas this did not occur in MERRF fibroblasts. However, it is also important to take into account that NAD^+^ and NADH are highly dynamic and unstable compounds, thus difficult to quantify in a reproducible way.

PGC-1α induction generated a greater and generalized boosting of mitochondrial biogenesis, however producing similar results in terms of respiration. Thus, it seems that increasing the absolute amount of wild type mtDNA as therapeutic strategy for MERRF syndrome is not as effective as initially predicted.

A different approach was the inhibition of mTORC1 through a low-dose treatment with rapamycin. mTORC1 is a key regulator of cellular metabolism, impinging multiple biological processes such as protein, nucleotide and lipid synthesis, autophagy and mitochondrial activity (Morita et al. 2015). Moreover, recent work demonstrated that mTORC1 orchestrates the mtISR in a mitochondrial myopathy model (Deletor mouse) and rapamycin administration was beneficial for this pathological condition (Khan et al. 2017; Forsström et al. 2019). A similar response has been obtained in a mouse model where myopathy was caused to defective Cox15 (Civiletto et al. 2018).

Dai et al. showed that a chronic treatment with low dosage of rapamycin (20 nM for 4 weeks) reduced the heteroplasmy of m.11778G > A mutation in cybrid cells (Dai et al. 2014). Using the same treatment, we obtained a significant improvement of mitochondrial respiration in MERRF fibroblasts, especially in the I-fibroblasts, which showed a complete recovery. This finding is in line with previous results obtained in different models of mitochondrial diseases, such as the Cox15 mouse model or MELAS fibroblasts (Civiletto et al. 2018; Cheema et al. 2021; Chung et al. 2021). The mechanism behind this beneficial effect may have implications beyond the autophagy stimulation expected following mTORC1 inhibition. In fact, the LC3-II levels increased exclusively in the I-fibroblasts, whereas were unchanged in both control and H-fibroblasts. Similarly, we reported only a modest reduction in the mtDNA heteroplasmy (5%), even if consistently in the same direction of increased wild-type. Differently, rapamycin produced a broad increment of OXPHOS protein levels, more pronounced in the I-fibroblasts than WT- and H-fibroblasts. This may be the result of boosted mitochondrial biogenesis, as also documented by TFAM amount, mtDNA content, NRF1 and mitochondrial gene expression, and it may be orchestrated by PGC-1α, since its gene expression was elevated by rapamycin. This positive modulation of rapamycin is in contrast with a few studies pointing on suppression of mito-biogenesis upon mTORC1 inhibition (Cunningham et al. 2007; Chaveroux et al. 2013; Morita et al. 2013). However, in line with our results, a recent study reported that hormetic doses of rapamycin could induce PGC-1α, Yin Yang 1 (YY1), TFAM and COX2 expression (Mahalakshmi et al. 2021). Increase of OXPHOS complexes have been also described in MELAS cells after chronic exposure to rapamycin (Chung et al. 2021).

Furthermore, it is documented that mTORC1 negatively regulates the activity of TFEB (Settembre et al. 2012; Martina et al. 2012), which in turn is able to modulate mitochondrial biogenesis both in a PGC-1α dependent or independent manner, by directly binding to the promoters of PGC-1α (Settembre et al. 2013) or NRF1 (Mansueto et al. 2017). Indeed, a few TFEB downstream targets (CTSD, LAMP1, BECLIN1 and p53) were upregulated by the chronic rapamycin treatment, especially in the I-fibroblasts that showed the best recovery in mitochondrial respiration.

The same concentration of rapamycin (20 nM) administered for a shorter time did not reproduce the same positive influence on mitochondrial respiration and biogenesis, and, interestingly, did not induce TFEB activation, as documented by unchanged gene expression of a few of its targets. Moreover, both acute and chronic rapamycin treatment were ineffective in rescuing the bioenergetics defect in MERRF cybrids, possibly because of the cancerous origin of this cell model.

These results support a possible role for TFEB-mediated mitochondrial and lysosomal biogenesis induction in the compensatory effect of rapamycin in MERRF fibroblasts. This implies that the global rapamycin mechanism of action is fairly complex and dose/time-dependent, not yet completely elucidated and most importantly, not limited to the assumption that is mainly driven only by mTORC1 inhibition promoting mitophagy.

## Conclusions

In conclusion, our study provides novel information on the potential use of rapamycin as beneficial therapy for MERRF syndrome, in addition to its suggested effectiveness for other mitochondrial diseases (Civiletto et al. 2018; Khan et al. 2017; Cheema et al. 2021; Chung et al. 2021; Johnson et al. 2013; Zheng et al. 2016; Wang et al. 2016). Nonetheless, since the effect of mTORC1 inhibition seems to have different consequences depending on cell type (Ignatenko et al. 2020), it remains crucial to test rapamycin treatment and reconfirm our current results in different MERRF models, such as neurons or myoblasts, which are targeted by the disease in patients.

## Supplementary Information

**Additional file 1.** Primer list and additional figures.

## Data Availability

All data generated or analyzed during this study are included in this published article.

## Abbreviations

MERRF: Myoclonus, epilepsy and ragged-red-fibers
RRFs: Ragged red fibers
mtDNA: Mitochondrial DNA
PGC-1α: Peroxisome proliferator-activated receptor-gamma coactivator 1 α
CPEO: Chronic progressive external ophthalmoplegia
OXPHOS: Oxidative phosphorylation
NDUFB8: NADH: Ubiquinone oxidoreductase subunit B8
COXII: Cytochrome c oxidase subunit II
CS: Citrate synthase
VDAC: Voltage-dependent anion-selective channel
TOM20: Translocase of outer mitochondrial membrane 20
SDHB: Succinate dehydrogenase complex iron sulfur subunit B
UQCRC2: Ubiquinol-cytochrome C reductase core protein 2
ATP5A: ATP Synthase F1 Subunit Alpha
TIM23: Translocase of inner mitochondrial membrane 23
NA: Nicotinic acid
NAD^+^: Nicotinamide adenine dinucleotide
SIRT1: Sirtuin 1
ATP6: Mitochondrially Encoded ATP Synthase Membrane Subunit 6
TFAM: Transcription Factor A, Mitochondrial
mTORC1: Mammalian target of rapamycin complex 1
MAP1LC3B, LC3B: Microtubule associated protein 1 light chain 3 beta
NRF1: Nuclear respiratory factor 1
TFEB: Transcription factor EB
CTSD: Cathepsin D
LAMP1: Lysosomal associated membrane protein 1
BECN1: Beclin 1
P53: Tumor Protein 53
OMM: Outer mitochondrial membrane
IMM: Inner mitochondrial membrane
mtUPR: Mitochondrial unfolded protein response
mtISR: Mitochondrial integrated stress response
FGF21: Fibroblast growth factor 21
GDF-15: Growth differentiation factor 15
MELAS: Mitochondrial myopathy, encephalopathy, lactic acidosis and stroke-like episodes
AMPK: AMP-Activate kinase alpha 1 subunit
Cox15: Cytochrome c oxidase assembly protein COX15 homolog
YY1: Yin Yang 1
TK: Thymidine kinase 1
SRB: Sulforhodamine B
OCR: Oxygen consumption rate
FCCP: Carbonylcyanide 4-(trifluoromethoxy) phenylhydrazone
RAPA: Rapamycin
