# Supplementary material for: Rapamycin rescues mitochondrial dysfunction in cells carrying the m.8344A > G mutation in the mitochondrial tRNALys
Source: Mol Med. 2022 Aug 3;28:90. doi: 10.1186/s10020-022-00519-z (PMC9347137; doi:10.1186/s10020-022-00519-z)
Supplement: Supplementary file 1 — Additional file 1. Primer list and additional figures. [file 10020_2022_519_MOESM1_ESM.docx]

**Additional file 1**

**Rapamycin rescues mitochondrial dysfunction in cells carrying the m.8344A>G mutation in the mitochondrial tRNA^lys^**

**List of content:**

Additional file 1_Figure S1

Additional file 1_Figure S1 legend

Additional file 1_Figure S2

Additional file 1_Figure S2 legend

Additional file 1_Figure S3

Additional file 1_Figure S3 legend

Additional file 1_Primer list

**Figure S1**

**
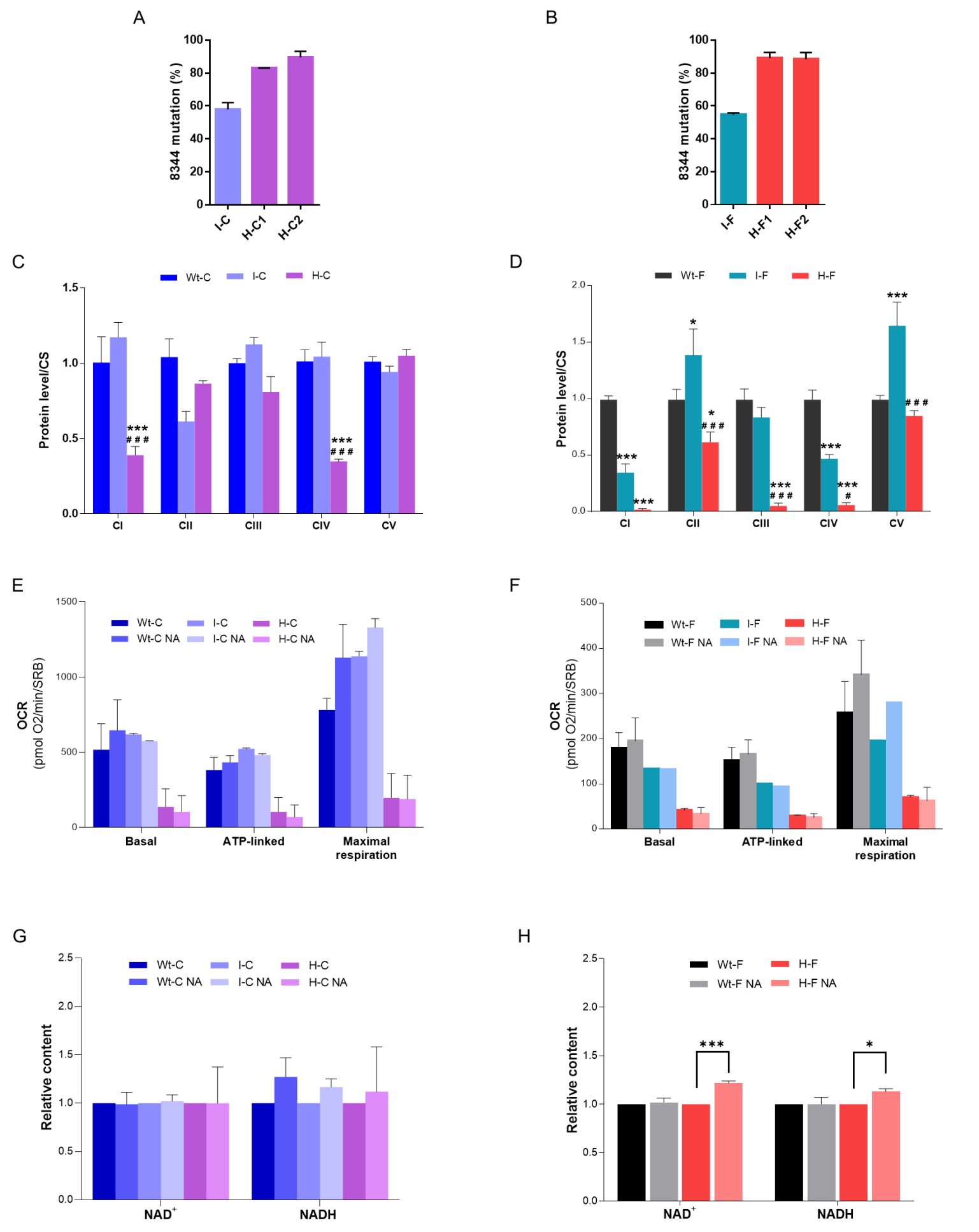
**

**Additional file 1_Figure S1 Legend**

**(A)** m.8344A>G mutation heteroplasmy level evaluation by SNaPshot method in the MERRF cybrid cell lines used in the study. Data are means + SD of two experiments. **(B)** m.8344A>G mutation heteroplasmy level evaluation by SNaPshot method in the MERRF fibroblasts used in the study. Data are means + SEM of six experiments. Densitometric analyses of OXPHOS proteins in cybrids **(C)** and fibroblasts **(D)**, under basal conditions and normalized to citrate synthase (CS) mitochondrial mass protein. All data are means + SEM of three independent experiments. Basal, ATP-linked and maximal respiration in cybrids **(E)** and fibroblasts **(F)** after 10 mM NA treatment for 96 h. Parameters were calculated from OCR traces and reported in the graph. In cybrids, data are means + SD of two experiments, analyzing two biological replicates. In fibroblasts, data are means + SD of three experiments for Wt-F and of two experiments for H-F; only one experiment could be performed in I-F due to spontaneous heteroplasmy shift in culturing cells. **(G-H)** NAD^+^ and NADH quantification in cybrids **(G)** and fibroblasts **(H)** after 10 mM NA treatment for 96 h. NAD^+^ and NADH levels (ng) were normalized on mg of protein in the sample and then expressed as ratio between treated cells and untreated, for each cell lines analyzed. Data are expressed as mean + SEM of three independent experiments.

Statistical analyses were performed with ANOVA test (Tukey’s multiple comparisons test) for **(C-F)**. * and *** values significantly different from the control cells, p <0.05 and p <0.001 respectively; ^#^ and ^###^ values significantly different from the I-mutant, p <0.05 and p <0.001, respectively. Wt-C, wild type cybrids; I-C, intermediate heteroplasmy cybrids; H-C, high heteroplasmy cybrids. Wt-F, wild type fibroblasts; I-F, intermediate heteroplasmy fibroblasts; H-F, high heteroplasmy fibroblasts. For **(G-H)**, unpaired two tail T-test was used to compare untreated *vs* NA treated cells. * p value <0.05, *** p value <0.001.

**Figure S2**

**
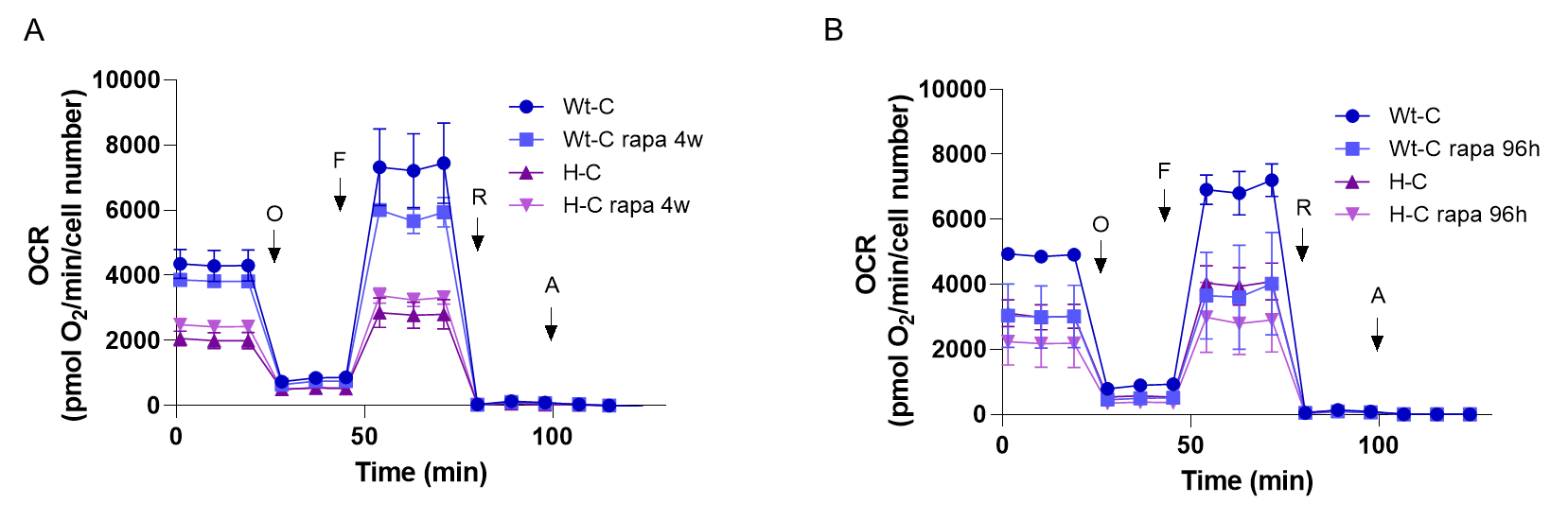
**

**Additional file 1_Figure S2 Legend**

OCR in cybrids after 4 weeks **(A)** or 96 hours **(B)** treatments with 20 nM rapamycin, expressed as pmoles O_2_/min normalized for cell number, under basal conditions and after injection of oligomycin (O), carbonyl cyanide 4-(trifluoromethoxy) phenylhydrazone (FCCP; F), rotenone (R) and antimycin A (AA). OCR traces show means ± SD of two experiments, analyzing two biological replicates. No significant differences were found comparing untreated and treated cell lines, in both conditions.

**Figure S3**

**
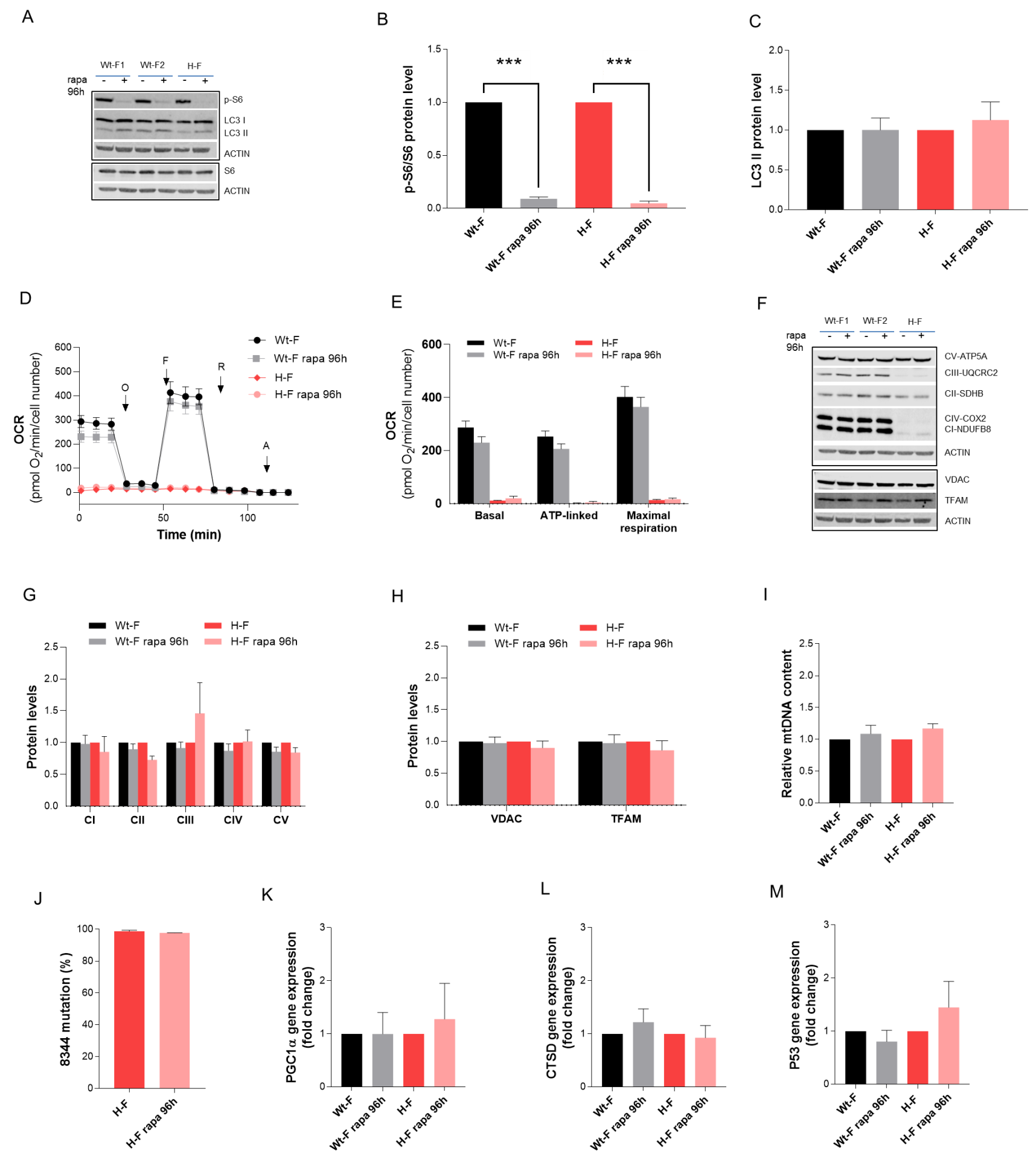
**

**Additional file 1_Figure S3 Legend**

Wt-Fibroblasts and H-Fibroblasts were treated with 20 nM rapamycin for 96 hours. **(A)** Representative Western Blot of p-S6, S6 and LC3 proteins. ACTIN was used ad loading control. **(B)** Densitometric analyses of the downstream target of mTORC1, p-S6 protein. **(C)** Densitometric analyses of LC3-II protein. **(D)** OCR expressed as pmoles O_2_/min normalized to cell number, under basal conditions and after injection of oligomycin (O), carbonyl cyanide 4-(trifluoromethoxy) phenylhydrazone (FCCP; F), rotenone (R) and antimycin A (AA). Data are shown as means **±** SEM of three independent experiments. **(E)** Basal, ATP-linked and maximal respiration were calculated from OCR traces and reported in the graph. **(F)** Western Blot analyses of OXPHOS proteins, VDAC and TFAM mitochondrial mass proteins. One representative experiment is shown. Densitometric analyses of OXPHOS **(G)** and VDAC and TFAM proteins **(H)**. **(I)** mtDNA content evaluation by qPCR. **(J)** m.8344A>G mutation heteroplasmy evaluation by SNaPshot method. **(K-M)** Gene expression of PGC-1α **(K)**, CTSD **(L)**, and p53 **(M)** evaluated by qPCR. ACTIN was used as reference gene.

If not specifically indicated, all data are normalized to untreated cells and are means + SEM of three biological replicates. Statistical analyses were performed using unpaired two-tail T-test to compare untreated *vs* treated cell lines. *** p <0.001.

**Additional file 1_Primer list**

**SNaPshot Method for m.8344A>G quantification**

| **Target** | **PCR primers** | **SNaPshot primer** |
| --- | --- | --- |
| **8344 F** | 5’ AACCAAACCACTTTCACCGC 3’ | 5’ TTTAATTAAAATTAAGAGA A 3’ |
| **8344 R** | 5’ ATGGGCTTTGGTGAGGGAGG 3’ |  |

**Gene expression analysis**

| **Target** | **Real Time PCR forward primers** | **Real Time PCR reverse primers** | **Universal Probe Library #** |
| --- | --- | --- | --- |
| **MT-ND1** | TGCGAGCAGTAGCCAAACAAT | TGATGGCAGGAGTAATCAGAGG |  |
| **MT-CO2** | ACAGATGCAATTCCCGGACGTC | TGGGCATGAAACTGTGGTTTGCTC |  |
| **MT-ATP6** | ATTCAACCAATAGCCCTGGCCG | ACGTAGGCTTGGATTAAGGCGAC |  |
| **PGC-1α** | ACGCACCGAAATTCTCCCTT | GGCGCTCTTCAATTGCCTTC |  |
| **P53** | CCCATCCTCACCATCATCACAC | TTGGGCAGTGCTCGCTT |  |
| **LAMP1** | AAGTACAACGTGAGCGGCA | CTTGTCACCGTCGTGTTGTC |  |
| **BECLIN** | ACGTGGAAAAGAACCGCAAG | CTCTCTGATACTGAGCTTCCTCC |  |
| **NRF1** | CCAGTCTCTGTGGACAAAATGA | GGCTTGCAGGCTTTCTTTCC | 64 |
| **CSTD** | CATCTTCTCCTTCTACCTGAGCA | GTCTGTGCCACCCAGCAT | 64 |
| **ACTB** | CCAACCGCGAGAAGATGA | CCAGAGGCGTACAGGGATAG | 64 |

**Cloning of PGC-1α into pLenti-DDK-P2A-Puro empty vector**

| **Name** | **Primer sequence** |
| --- | --- |
| **PGC1-α Fw:** | TCATGGCGCGCCATGGATGAGACCTCCCCAAGGCTGG |
| **PGC1-α Rv:** | CGATCTCGAGCCTGCGCAAGCTTCTCTGAGCTTC |
